# The Futility of Nugent Scoring as a Diagnostic Tool for Neovaginal Bacterial Dysbiosis in Transfeminine People

**DOI:** 10.21203/rs.3.rs-7428168/v1

**Published:** 2025-09-08

**Authors:** Jessica Prodger, Reeya Parmar, Bern Monari, Emery Potter, Jorge Rojas-Vargas, Hannah Wilcox, David Zuanazzi, Annabel Poon, Ainslie Shouldice, Vonetta Edwards, Yonah Krakowsky, Jacques Ravel

**Affiliations:** Western University; University of Western Ontario; University of Western Ontario; University of Western Ontario; University of Western Ontario; University of Western Ontario; University of Western Ontario; University of Maryland, Baltimore; University of Maryland, Baltimore; University of Maryland, Baltimore; University of Maryland, Baltimore; University of Maryland, Baltimore

**Keywords:** Bacterial Vaginosis, Gynecology, Microbiome, Transgender, Vaginoplasty

## Abstract

**Background:**

Transfeminine people were assigned male at birth and experience a female or feminine gender identity. Many elect to undergo vaginoplasty, a surgical procedure that constructs a neovagina, typically using penile and scrotal tissue. Like cisgender females, transfeminine people experience gynecological symptoms, including pain, discharge, and malodor. In cisgender females, clinicians attribute these symptoms to bacterial dysbiosis and can be diagnosed by Nugent scoring of gram-stained vaginal smears. The Nugent score assesses the abundance of large gram-positive rod vs. small or curved gram-variable rod morphotypes, traditionally for the detection of *Lactobacillus*spp., *Gardnerella vaginalis*, and *Mobiluncus* spp. (curved rod), respectively. Although unvalidated for neovaginal samples, this method is frequently applied to diagnose dysbiosis in transfeminine people with vaginoplasty.

**Objective:**

This study assessed the Nugent score’s utility as a clinical tool for diagnosing neovaginal dysbiosis in transfeminine people who underwent penile inversion vaginoplasty.

**Study Design:**

As a part of the TransBiota study, n=39 transfeminine participants self-collected neovaginal smears. Smears were Gram stained and Nugent scored, and Nugent scores were correlated with existing data on neovaginal bacterial composition (16S rRNA gene sequencing), neovaginal cytokines (Luminex multiplex immunoassay), and self-reported symptoms.

**Results:**

More than 70% of smears fell in the 7–10 range that would indicate Bacterial Vaginosis in cisgender women. However, Nugent score failed to correlate with the abundance of Nugent-targeted bacteria. Bacteria with similar morphotypes, but not belonging to *Lactobacillus*, *Gardnerella*, or *Mobiluncus*, were highly abundant and prevalent in the neovagina. Nugent score also failed to predict local inflammation or clinical symptoms.

**Conclusion:**

The Nugent score is not an effective tool to identify neovaginal dysbiosis or indicators of health in transfeminine individuals. Clinicians need the development of accurate, evidence-based diagnostic tools for the neovagina.

## INTRODUCTION

Transfeminine individuals (TF) were assigned male at birth and experience a female/feminine gender identity. Numerous communities fall under this umbrella, including transgender women, non-binary, and other gender diverse individuals^1,2^. Many TF elect to undergo gender-affirming medical care, often through feminizing hormone therapy or surgery. Vaginoplasty is a widely performed gender-affirming surgery that creates a clitoris, vulva, and vaginal canal. The most prevalent technique is penile inversion vaginoplasty, involving orchiectomy, dissection of the space between the bladder and rectum, and lining the newly formed space with penile and scrotal tissue^3^. Although less common, sigmoid or peritoneal tissue may also be used to augment the vaginal canal^4–6^. Penile inversion vaginoplasty results in a vaginal canal that is visually and functionally similar to that of cisgender females (CF) but lined with soft-cornified skin. We use the term “vagina” to refer to the vaginal canal of those born with a vagina, and “neovagina” to refer to the surgically created vaginal canal of TF. Our aim in using two terms is to clinically distinguish between vaginal canals lined with epithelia of different origins.

In 2021, an estimated 20% of TF in the US had undergone genital surgery, with an additional 67% desiring it^7^. Like CF, TF with vaginoplasty often experience genital symptoms, including itching, burning, discharge, and malodor; however, the source of these symptoms has not been explored. To address this gap, we performed a study of the neovaginal microenvironments of 47 TF living in Canada^8,9^. In this study, 56% of participants reported neovaginal symptoms within the past 30 days^10^. In reproductive-aged CF (rCF), similar symptoms are caused by bacterial vaginal dysbiosis termed “bacterial vaginosis” (BV), which occurs when beneficial *Lactobacillus* spp. are replaced by a polymicrobial microbiome, including *Gardnerella vaginalis*, *Prevotella* spp., *Ca*. Lachnocurva vaginae, and other anaerobic bacteria^11–13^. *Lactobacillus* spp. play a critical role in rCF vaginal health by producing antimicrobial compounds and lactic acid, which lowers pH and inhibits colonization by pathogens^14,15^. Even without symptoms, dysbiotic microbiomes in rCF are associated with increased susceptibility to sexually transmitted infections, including chlamydia, gonorrhea, HSV-2, and HIV-1, underscoring the importance of the vaginal microbiome in sexual health^14–23^.

Clinicians usually diagnose BV in rCF using the Nugent score. The Nugent score is a Gram stain-based diagnostic measure that rates the relative abundance of gram-positive rods (indicative of beneficial *Lactobacillus* spp.) vs. small or curved gram-variable rods (morphotypes of BV-associated bacteria)^12,24^. Scores from 0–3 are considered optimal, 4–6 inconclusive or intermediate, and 7–10 indicative of BV. The Nugent score is often applied to diagnose TF experiencing genital symptoms; however, the neovaginal microbiota is distinct from the vaginal microbiota^25–27^, and results from TransBiota suggest bacteria associated with inflammation in the neovagina differ significantly from those in the rCF vagina^8^. Bacteria associated with neovaginal immune activation include *Lawsonella*, *Howardella*, *Fusobacterium*, and *Parvimonas*, while higher abundances of *Ezakiella*, *Fastidiosipila*, *Murdochiella*, and *Peptoniphilus* associated with reduced inflammation^8^. Core neovaginal bacteria are listed in **Supplemental Table 1**.

The substantial differences in microbiome composition and associated inflammation found in the rCF vagina and the neovagina raise questions about the utility of the Nugent score as a diagnostic tool in TF. Nevertheless, clinicians lack alternative diagnostics and the Nugent score continues to be frequently employed^28^. This study evaluated the Nugent score’s relevance as a diagnostic tool for neovaginal dysbiosis by assessing its accuracy in identifying bacteria targeted by the score, and its ability to predict markers of bacterial dysbiosis (cytokines and symptoms) in the neovagina.

## METHODS

### Participants

TransBiota was a study investigating the genital microenvironments of trans and other gender diverse people receiving gender-affirming medical care^8–10^. Eligible TF participants were Canadian residents 18 years or older, who underwent vaginoplasty > 1 year prior to study entry. Research ethics board approval was obtained from Western University (REB #115503) and University of Maryland (IRB #HP-00096952), and all participants provided written informed consent. Participants were recruited online through social media and community groups, healthcare provider referral, and re-contact of consenting Trans PULSE Canada participants^29^. Participants were mailed a study kit containing instructions and self-collection materials. Demographic, behavior, and symptom data were collected through an online questionnaire. Participants (n = 47) returned three weekly neovaginal sample sets by mail using pre-paid envelopes. This analysis includes the first timepoint for each participant for which there is a corresponding 1) scorable gram-stained slide, 2) microbiome data, and 3) immune analyte data.

All research activities were conducted in accordance with institutional and ethical regulations, and participant data were deidentified prior to analysis.

### Microbiota and Cytokine Analyses

Participants self-collected neovaginal swabs at each timepoint for Nugent scoring, microbiota analysis, and cytokine quantification. Sample collection and analysis of neovaginal microbiota and cytokines have been described in detail elsewhere^8^. In brief, participants were instructed to insert each swab (Puritan HydraFlock) 5cm into the neovaginal canal, rotate 3 times, and place in collection media. Swabs for microbiota analysis were collected into 1ml of Qiagen C2.1 solution. DNA extractions were performed using the MagAttract PowerMicrobiome DNA/RNA kit (Qiagen), and 16S rRNA gene V3-V4 region amplicon sequencing (amplified via two step PCR) was conducted at University of Maryland Institute for Genomic Sciences^30^. Reads were processed using a QIIME-dependent script with DADA2 to generate amplicon-sequence variants (ASVs). Taxonomy was assigned with the RDP naïve-Bayes classifier trained on the SILVA v138.2 16S rRNA database and refined species-level calls with SpeciateIT v2.0.0. ASVs sharing identical taxonomy were collapsed. Swabs for cytokine analysis were collected into 500μl of a PBS-based stabilizing buffer, and IL-1a, IL-1b, IL-6, IL-8, MIG, MIP-1b, and RANTES concentrations were quantified on a Luminex MAGPIX system.

### Nugent Scoring

Participants rolled collection swabs onto charged glass microscope slides (USA Scientific) and returned them to Western University in secured plastic cases. Neovaginal smears were heat-fixed and Gram stained using standard techniques. In brief, slides were stained with crystal violet (1 minute), iodine mordant (1 minute), decolorizing solution (until solution ran clear), and safranin (30 seconds) at room temperature. Slides were rinsed with running tap water between steps. Slides were observed under a 100x oil-immersion light microscope. For each smear, bacterial morphologies were individually scored in 10 representative fields of view (FOVs) following Nugent criteria^24^. The criteria assign a decreasing score from 0–4 for the abundance of *Lactobacillus*-like gram-positive large rods, an increasing score from 0–4 for the abundance of *Gardnerella*-like short gram-variable straight rods, and an increasing score from 0–2 for the abundance of *Mobiluncus*-like gram-variable curved rods. Scores for each morphotype were summed to yield a total score from 0–10 for each FOV. Final mean score across the 10 FOVs was calculated for each participant. In rCF, scores of 0–3 are optimal, 4–6 inconclusive/intermediate, and 7–10 indicative of BV^24^.

### Data Analysis

Multiple unpaired Mann-Whitney U nonparametric tests were used to assess differences in Nugent scores between asymptomatic and symptomatic participants. Fisher’s exact test was used to assess differences in bacterial prevalence, and Mann-Whitney U test was used to assess differences in relative abundance between asymptomatic and symptomatic participants. Associations between Nugent score and cytokines (IL-1α, IL-1β, IL-6, IL-8, MIG, MIP-1β, and RANTES) were assessed by Spearman’s correlation. Relationship between neovaginal bacteria taxa and cytokines, as well as bacterial taxa and Nugent score was also evaluated using Spearman’s correlations. Only taxa with a neovaginal prevalence of > 25% were included in analyses. GraphPad Prism 8 and R Studio (version 4.3.2) were used to create graphs and perform statistical analyses.

## RESULTS

Of the n = 47 TransBiota participants, 8 were excluded (17%) due to no scorable slides from destruction during heat-fixation from smearing on the incorrect side of the slide; damage to slides during shipping; or insufficient sample for Nugent scoring (defined at < 30 bacteria/FOV). Demographics on the remaining n = 39 participants included in this study are displayed in Table 1. Median time on hormone therapy was 5.6 years and median time since vaginoplasty 2.8 years.

**Figure 1:**
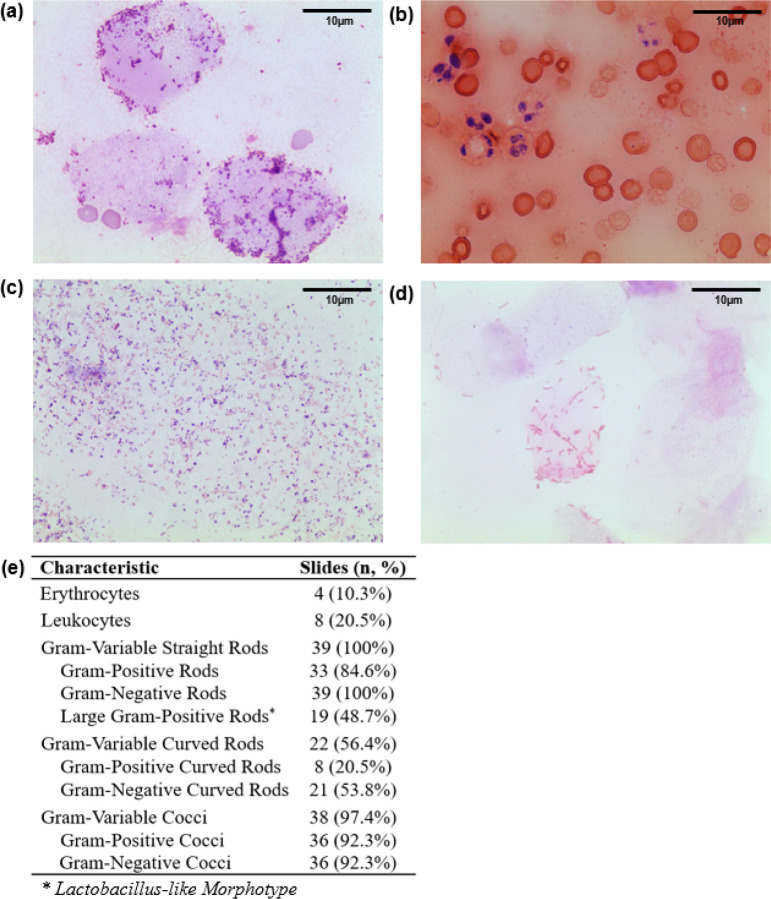
Representative images and descriptive characteristics of neovaginal smears. Self-collected neovaginal smears (n=39) were heat-fixed, Gram-stained, and observed under a 100x oil-immersion light microscope. Ten fields of view (FOV) were scored per participant; representative FOVs showing **(a)** proximity of bacteria to epithelial cells, **(b)** abundance of red and white blood cells, **(c)**dominance with Gram-variable rods and cocci, and **(d)** dominance with *Lactobacillus*-like morphotype. Descriptive characteristics of neovaginal smears are shown in **(e)**.

Neovaginal smears contained nucleate and anucleate epithelial cells, with most bacteria clustered near these epithelial cells (Fig. 1a, e). Four slides (10.3%) were visibly bloody with erythrocytes and leukocytes visible by microscopy (Fig. 1b, e). Gram-variable rods and cocci dominated the majority of smears (Fig. 1c, e). Although all smears contained gram-variable straight rods, only 48.7% of slides displayed large gram-positive rods (*Lactobacillus*-like morphotype), and just three (7.7%) were dominated by large gram-positive rods (Fig. 1d, e).

**Figure 2:**
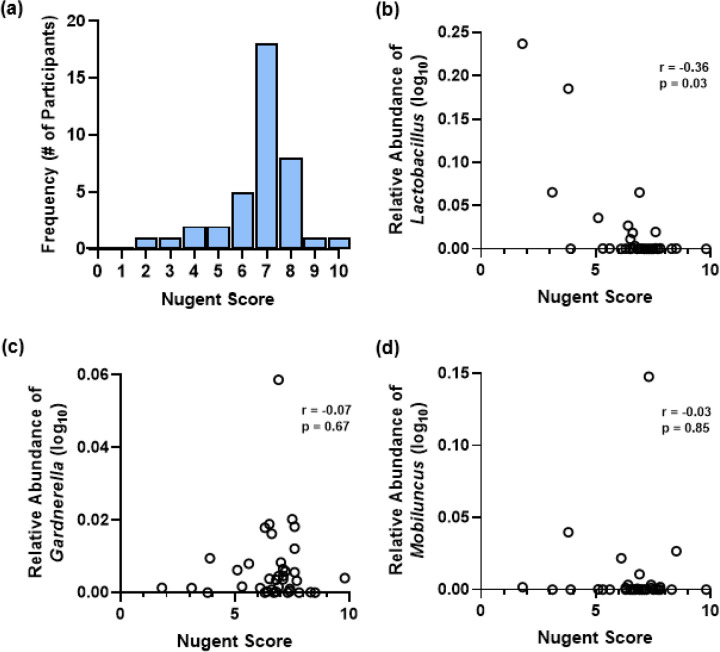
Nugent scores and correlation with traditional Nugent-targeted taxa across neovaginal smears. **(a)** Distribution of Nugent scores from n=39 TF participants. Scores represent the median score per participant over 10 fields of view (FOVs) acquired at 100x oil-immersion magnification. Lower scores from 0–3 are considered optimal, intermediate scores of 4–6 are inconclusive, and higher scores of 7–10 are considered indicative of BV. Spearman’s correlations are shown between the relative abundances of **(b)** *Lactobacillus* (p<0.03), **(c)** *Gardnerella* (p=0.67), **(d)** and *Mobiluncus* (p=0.85) within neovaginal smears and Nugent score.

The majority of neovaginal smears (71.8%) fell within the BV Nugent range (7–10; Fig. 2a). The most common score was 7 (46.2% of participants) indicating abundant gram-variable rods and minimal *Lactobacillus*-like rods. Only two participants (5.1%) scored an optimal Nugent score range for rCF (0–3). *Lactobacillus* showed a moderate negative correlation with Nugent score (Fig. 2b) while *Gardnerella* and *Mobiluncus* showed no correlation (Fig. 2c, d).

### Nugent Score and traditional Nugent-targeted taxa are not associated with neovaginal symptoms

**Figure 3:**
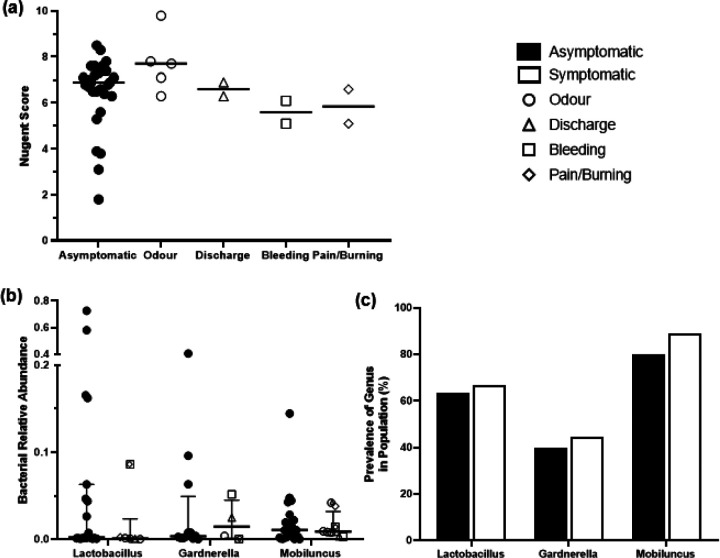
No differences in Nugent score or traditional Nugent score bacteria between symptomatic and asymptomatic participants. **(a)** Median Nugent score did not vary between asymptomatic participants and participants who self-reported neovaginal malodor, discharge, bleeding, or pain/burning in the last 7 days (Mann-Whitney U, asymptomatic participants vs. each symptom, all p>0.1). Comparison of the **(b)** prevalence and **(c)**relative abundance of *Lactobacillus*, *Gardnerella*, and *Mobiluncus*between asymptomatic and symptomatic participants (prevalences compared by Fisher’s exact test, relative abundances by Mann Whitney U, all p>0.8). Median and interquartile range are shown.

Nine participants (23.1%) reported neovaginal symptoms (malodor, discharge, bleeding, pain/burning) within 7 days prior to sample collection. There was no difference in Nugent scores between symptomatic and asymptomatic participants (Fig. 3a). Additionally, no relationship was observed between prevalence (Fig. 3b) or proportional abundance (Fig. 3c) of the traditional Nugent score bacteria (*Lactobacillus*, *Gardnerella*, *Mobiluncus*) and the presence or absence of neovaginal symptoms.

### Nugent Score is not associated with neovaginal cytokines

**Figure 4:**
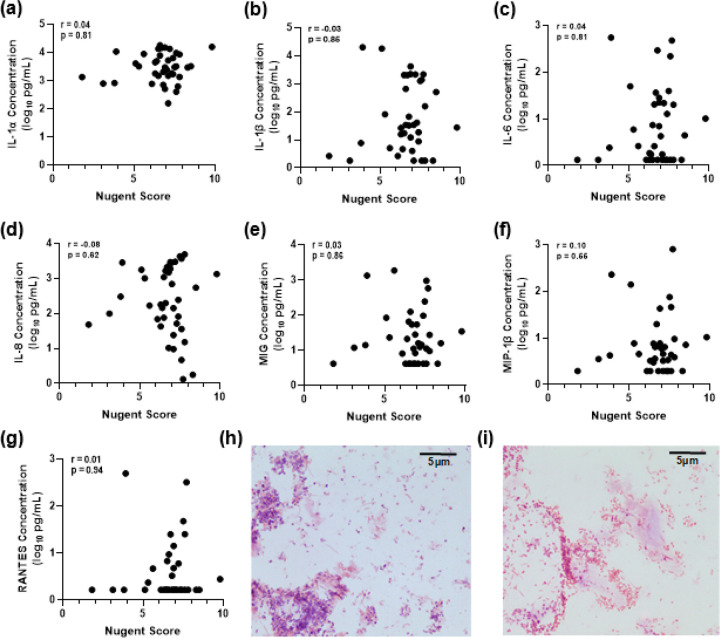
Neovaginal cytokines do not correlate with Nugent score. Self-collected neovaginal smears from transfeminine participants (n=39) were heat-fixed, Gram stained, and observed under a 100x oil-immersion light microscope. Cytokine concentrations in neovaginal swabs were measured by multiplex immunoassay (Luminex). Spearman’s correlations were used to assess associations between Nugent score and **(a)** IL-1α, **(b)**IL-1β, **(c)** IL-6, **(d)** IL-8, **(e)** MIG, **(f)** MIP-1β, and **(g)** RANTES. Representative images of gram-stained neovaginal smears from participants with low (**h**) and high (**i**) cytokine levels. Images have been cropped for visualization.

Nugent scores were analyzed in relation to pro-inflammatory cytokine concentrations, but no significant correlations were observed (Fig. 4a – g). Representative images are shown of gram-stained smears from one participant with high cytokine levels (87.8pg/ml IL-1α, 65.4pg/ml IL-1β, 34.7pg/ml IL-6, 85.3pg/ml IL-8, 61.1pg/ml MIG, 21.7pg/ml MIP-1β, 33.1pg/ml RANTES) and one participant with low cytokine levels (83.9pg/ml IL-1α, 35.6pg/ml IL-1β, 6.3pg/ml IL-6, 57.1pg/ml IL-8, 11.8pg/ml MIG, 21.7pg/ml MIP-1β, undetectable RANTES). Both participants had a Nugent score of 7 with neovaginal smears dominated by gram-negative rods (Fig. 4h – i).

### Bacteria with Nugent-targeted morphotypes in the neovagina

**Figure 5:**
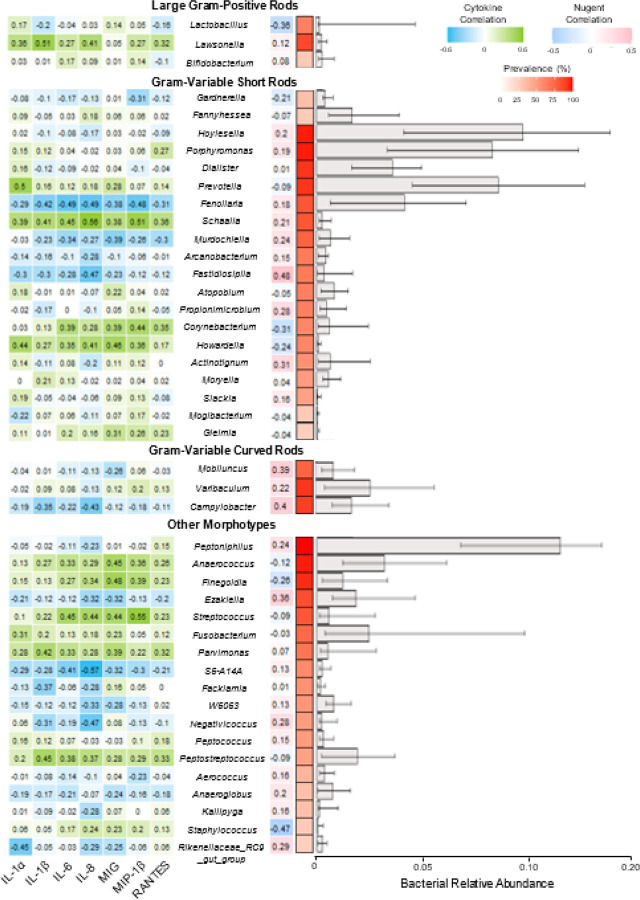
Neovaginal bacterial prevalence, relative abundance, and association with cytokines and Nugent score. Taxa typically scored by Nugent criteria (*Lactobacillus*, *Gardnerella*, *Fannyhessea*, and *Mobiluncus*) at the start of each morphotype category. Bacterial taxa with a prevalence of >25% are included. Median relative abundance is measured among prevalent participants and interquartile range are shown. Bacterial prevalence and relative abundance were measured from neovaginal swabs (n=39) using 16S rRNA gene sequencing. Cytokine concentrations were measured from swab eluent by multiplex immunoassay (Luminex). Spearman’s correlations were used to assess associations between taxa and IL-1α, IL-1β, IL-6, IL-8, MIG, MIP-1β, RANTES and Nugent score. Spearman’s correlations p values are shown in **Supplemental Table 3**.

A comprehensive description of neovaginal bacterial communities of TransBiota participants has been previously published^8^. A summary of the most prevalent neovaginal bacteria (> 25% prevalence) are listed in Fig. 5, separated by the morphotypes targeted by Nugent scoring.

Traditional Nugent-targeted taxa (*Lactobacillus*, *Gardnerella*, and *Mobiluncus*) frequently appeared in the neovagina, but at low relative abundance (Fig. 5). Several other taxa detected in the neovagina had similar morphologies to Nugent-targeted genera. *Lawsonella clevelandensis* is a large gram-positive rod that can have similar appearance to *Lactobacillus* spp. on Gram staining^31,32^. *Lawsonella clevelandensis* (87.2% prevalence; 0.3% median abundance) outnumbered *Lactobacillus* (64.1%; 0.2%). *Lawsonella* abundance positively correlated with IL-1α, IL-1β, IL-8 and RANTES (p < 0.05), whereas *Lactobacillus* showed no correlation with cytokines, and correlated with lower Nugent scores (Fig. 5; p < 0.03). Three participants exhibited predominantly large rods; two had low Nugent scores of 2 (72.6% *Lactobacillus* relative abundance) and 3 (58.0% *Lactobacillus* relative abundance) and one scored 4 (16.5% *Lactobacillus* relative abundance), owing to mixed morphotypes.

*Gardnerella vaginalis*, was detected in 33.3% of samples at a median relative abundance of 0.4%. *Fannyhessea vaginae* (formerly *Atopobium vaginae*), although not a traditional Nugent bacteria, is also a BV-associated rod and may contribute to the diagnostic power of the Nugent score in rCF^33^. *Fannyhessea* was detected in 28.2% of samples at a median relative abundance of only 1.7%. Short straight rods in the neovagina were more likely to be *Hoylesella* (previously Prevotella; 97.4% prevalence, 9.7% relative abundance), *Prevotella* spp. (92.3%; 8.5%), or *Porphyromonas* spp. (97.4%; 8.3%). *Prevotella* was positively associated with increased cytokines (IL-1α; p < 0.01), while *Gardnerella, Fannyhessea, Porphyromonas*, and *Hoylesella* had no significant correlation. None had significant correlations with Nugent score.

*Mobiluncus*, the gram-variable curved rod traditionally targeted by Nugent scoring, was detected in 76.9% of samples at 0.8% median relative abundance. Other abundant neovaginal curved rods included *Varibaculum* (92.3% prevalence, 2.6% abundance) and *Campylobacter* (89.7%; 1.7%). Abundances of *Campylobacter* were inversely correlated with neovaginal cytokines (IL-1β, IL-8; p < 0.05) while *Mobiluncus* and *Varibaculum* showed no significant correlation with cytokines. Both *Mobiluncus* (p < 0.02) and *Campylobacter* (p < 0.02) correlated with higher Nugent scores.

Of note, in addition to *F. vaginae*, BV-associated *Ca*. Lachnocurva vaginae (formerly BVAB1; curved rod) and *Sneathia amnii* (curved Gram-negative rod) posses similar morphotypes to traditional Nugent bacteria and may contribute to the diagnostic power of the Nugent score in rCF^34^. However, both were absent from neovaginal samples.

As noted in Fig. 1E, many neovaginal smears contained high abundance of cocci, which are not considered by Nugent criteria. Based on V3-V4 16S rRNA gene sequences, 4/10 core neovaginal bacteria have morphotypes not considered during Nugent scoring (**Supplemental Table 1**). *Peptoniphilus*, *Ezakiella* and *Anaerococcus* are all gram-positive cocci. Of these, *Ezakiella* is associated with reduced neovaginal cytokines (IL-8, MIG, p < 0.05), while *Anaerococcus* is associated with increased cytokines (IL-6, MIG, MIP-1β; p < 0.05) (Fig. 5).

## DISCUSSION

### Principal findings

This study provides strong evidence that the Nugent score is not suitable for clinical diagnosis of neovaginal dysbiosis in TF with penile inversion vaginoplasty. Most neovaginal bacteria belong to taxa not targeted by the Nugent score, and the score does not associate with predictors of genital dysbiosis, including neovaginal symptoms and cytokines.

Nugent scoring in rCF relies on the eubiotic nature of *Lactobacillus* spp. predominance, and vaginal polymicrobialism as dysbiotic. However, *Lactobacillus* spp. predominance in the neovagina is very rare^8,27,28,35^, potentially due to differences in carbon sources available in the rCF vagina and TF neovagina. Vaginal epithelial cells in rCF are rich in glycogen and constantly shed into the vaginal lumen^15,36,37^. In contrast, neovaginal epithelium derived from penile skin lacks glycogen despite exposure to estrogen levels similar to rCF, and is instead soft-cornified with a lipid-rich extracellular matrix^15,36–39^. In rCF, vaginal *Lactobacillus* spp. metabolize glycogen products to produce lactic acid with anti-inflammatory properties, inhibiting colonization by non-lactobacilli, including pathogens^40,41^. In our cohort *Lactobacillus* rarely dominated and showed similar abundance in symptomatic and asymptomatic TF. Future research is warranted to determine if the neovaginal epithelium can support *Lactobacillus* predominance, and if this confers any benefit.

Further, there is no evidence that neovaginal microbiomes rich in gram-variable rods are necessarily dysbiotic. Neovaginal abundances of traditional Nugent morphotypes *Gardnerella* and *Mobiluncus* were not different between symptomatic and asymptomatic TF and did not correlate with increased cytokines. While the relationship between neovaginal inflammation and sexual health outcomes has not been adequately explored, genital inflammation is strongly correlated with negative health outcomes in cisgender individuals^16,26,42–45^. In rCF, *Mobiluncus* and *Gardnerella* are positively associated with vaginal cytokines such as IL-1β and IL-8^42,46–48^. Instead in the neovagina, *Campylobacter*, a more abundant curved rod, showed negative correlations with cytokines. More abundant short gram-variable rods such as *Hoylesella* (predominantly *H. timonensis* and *H. buccalis*, both previously *Prevotella*), *Porphyromonas* and *Dialister* lacked any association with inflammation, while *Fenollaria* correlated inversely with cytokines. Other bacteria that normally contribute to the diagnostic power of the Nugent score in rCF (*Fannyhessea*, *Sneathia amnii*, *Ca*. L. vaginae) showed no correlation to cytokines or were absent from the neovagina. These data suggest that gram-variable rod abundance on neovaginal smears does not provide interpretive information on neovaginal health.

*Lawsonella* abundance correlated strongly with inflammation, yet Nugent scoring classifies *Lawsonella* morphotypes under the “beneficial rod” category. Likewise, pro-inflammatory cocci such as *Anaerococcus* lay outside the Nugent framework. These findings are consistent with a study by Weyers et al. examining neovaginal cytology, reporting that, despite over 50% of participants being diagnosed with BV, no correlation was observed with neovaginal inflammation^49^. Additionally, Weyers et al. noted a significant presence of inflammatory cells in the neovagina. In our study, we frequently observed blood cells, suggesting participants may be experiencing epithelial erosion.

### Clinical implications

Nugent scoring of neovaginal smears risks misdiagnosis and promotes futile antibiotic use. Nearly all neovaginal smears in this study fell outside the optimal Nugent range (0–3), indicating most would be classified as dysbiotic and indicative of BV. BV in rCF is commonly treated with metronidazole, which spares *Lactobacilli* but is bactericidal for BV-associated gram-negative anaerobes, including *Gardnerella*^50,51^. However, the gram-positive bacteria associated with inflammation in the neovagina, such as *Lawsonella* and *Anaerococcus*, are unlikely to be susceptible to metronidazole^50,51^. TF who receive a Nugent score in the BV range may feel distress or use products or home remedies aimed at treating BV and restoring *Lactobacilli* in rCF. TransBiota participants who used diverse solutions for douching (povidone-iodine, soapy water, vinegar) were more likely to have high abundances of inflammation-associated bacteria^8^. Many also reported using oral probiotics or probiotic suppositories designed for rCF when experiencing neovaginal symptoms, and referred to having BV in their questionnaires^10^. Additional research is urgently needed to better characterize the causative agents of neovaginal inflammation and symptoms, and to design effective diagnostic tools to identify them in a clinical setting. However, these findings apply chiefly to mature penile-skin lined neovagina, and microbial dynamics might differ in bowel-segmented or peritoneal graft neovaginas.

### Limitations

This study’s mail-in methodology enabled broader participation, but resulted in unusable smears due to inadequate sampling or slide damage during shipping. This study also did not address sampling location within the neovaginal canal; while participants were instructed to collect swabs 5cm into the neovaginal canal, sampling depth may have varied between participants and microbiomes vary by distance from the introitus. Although participants were asked to refrain from inserting anything into their neovagina 24h prior to sampling, variable practices outside this window may have introduced variability^52,53^. Unmeasured behavioral or hormonal variables may also confound associations.

### Conclusions

The Nugent score is an ineffective tool for predicting neovaginal dysbiosis in TF with penile inversion vaginoplasty. Bacteria traditionally targeted by the Nugent score are rare in the neovagina, while other taxa with similar morphotypes are abundant. Nugent scores did not correlate with inflammation or symptoms in the neovagina. Using the Nugent score on neovaginal smears may result in misdiagnosis, inappropriate antibiotic use, and misplaced efforts by TF and clinicians to “correct” neovaginal microbiomes, possibly disrupting an optimal microbiome. These findings highlight that vaginal dysbiosis differs fundamentally between rCF and TF and underscores the need to establish evidence-based neovaginal diagnostics.

## Supplementary Files

This is a list of supplementary files associated with this preprint. Click to download.

- NugentScoreData.xlsx
- MicrobiomeData.xlsx
- CytokineData.csv
- SUPPLEMENTALMATERIAL.docx

## Figures and Tables

**Table 1:** Participant demographics.

	Participants (n = 39)
Age, years (median, range)	39 (26–67)
Ethnoracial Identity (%)	
White	89.7%
Latin American	2.6%
Jewish	2.6%
Mixed Ethnicity	5.1%
Months on hormone therapy (median, range)	67 (35–265)
Months since vaginoplasty (median, range)	33 months (12–229 months)
Circumcised prior to vaginoplasty (%)	56.4%
Symptoms, past 7 days (%)	23.1%
Bleeding	5.1%
Discharge	5.1%
Itching/burning	2.6%
Malodor	12.8%
Pain	2.6%
pH (median, range)	5.5 (4.5–8)

## Data Availability

All source codes used to analyze the data and generate the figures presented are available in GitHub at github.com/prodgerlab/TransBiota/tree/main/Nugent_scoring_paper. The cytokine, microbiome, and Nugent scoring datasets used in this study are also accessible through this repository.
